# Clinical Characteristics and Treatment Courses of Trauma-Induced Thrombotic Microangiopathy: A Retrospective Study

**DOI:** 10.3390/jcm13216527

**Published:** 2024-10-30

**Authors:** Suyeong Hwang, Gun Woo Kim, Sung Hoon Cho, Kyoung Hoon Lim

**Affiliations:** Department of Surgery, Trauma Center, Kyungpook National University Hospital, School of Medicine, Kyungpook National University, Daegu 41944, Republic of Korea; tndud4857@knu.ac.kr (S.H.); dryrain@knuh.kr (G.W.K.); chossis@knu.ac.kr (S.H.C.)

**Keywords:** thrombotic microangiopathy, trauma, acute renal failure, therapeutic plasma exchange, sepsis

## Abstract

**Introduction:** Thrombotic microangiopathy (TMA), defined by thrombocytopenia, microangiopathic hemolytic anemia, and organ injury, is not widely recognized as being trauma-related. This study aimed to describe the clinical features and outcomes of trauma-induced TMA (t-TMA) to assist in early diagnosis and management. **Methods:** A retrospective review was conducted on 30 trauma patients diagnosed with t-TMA between 2014 and 2019. Demographic, clinical, and laboratory data, as well as treatment methods, were analyzed. **Results:** Thrombocytopenia (<50,000/µL) occurred, on average, 2.9 days post-trauma, with diagnosis following 3.6 days later. Patients had a mean age of 67.6 years, and 63.3% were male. Clinical presentations included acute kidney injury (AKI) requiring renal replacement therapy (86.7%), altered mental status (53.3%), non-infectious fever (50%), and digital necrosis (43.3%). Eighteen patients were treated with therapeutic plasma exchange (TPE) alone, nine with TPE and methylprednisolone, and three with methylprednisolone alone. Remission was achieved in 96.7% of all cases. The mean TPE duration was 6.1 days, prolonged by prior platelet transfusions. The mortality rate was 26.7% (8/30), with sepsis being the most common cause of death (five patients), particularly for those treated with TPE and methylprednisolone. **Conclusions:** Trauma-induced TMA should be suspected in trauma patients presenting with unexplained thrombocytopenia, AKI, and elevated LDH. Early diagnosis and prompt treatment are crucial, while unnecessary platelet transfusions should be avoided. Careful infection management is critical to improving patient outcomes, particularly if patients are treated with TPE and methylprednisolone.

## 1. Introduction

Thrombotic microangipathies (TMAs) are characterized by microangiopathic hemolytic anemia, thrombocytopenia, and organ ischemic injury [1]. The pathologic features comprise vascular damage, which is visible as arteriolar and capillary thromboses, as well as characteristic abnormalities in the endothelium and vessel walls. Although rare, etiologies for TMA vary and are named depending on the cause [2]. However, trauma is never mentioned as one of the causes of TMA, and relatively few studies have reported incidences of TMA after trauma [3,4]. We documented many cases of patients at our hospital who experienced TMA after severe trauma, and their clinical courses were similar to those of patients with thrombotic thrombocytopenic purpura (TTP), as a disintegrin and metalloproteinase with thrombospondin motifs 13 (ADAMTS13)-deficiency-mediated TMA. However, as none of these patients had ADAMTS13 deficiency, as per the definition of TTP, we categorized these cases as “trauma-induced TMA” (t-TMA).

Thrombocytopenia is a common finding in patients with severe trauma due to coagulopathy caused by bleeding, although symptoms usually improve after hemostasis is completed and coagulopathy is resolved. In patients with severe trauma, thrombocytopenia can persist despite the absence of persistent bleeding. In these cases, physicians will often continue with platelet transfusion as they will be uncertain as to whether bleeding is continuing or whether it might recur. However, if persistent thrombocytopenia is caused by t-TMA rather than by consumptive coagulopathy following continuous bleeding, conventional treatments such as platelet transfusion can adversely affect the prognosis by aggravating the disease’s progress. The treatment strategies are entirely distinct and contingent upon the underlying causes of thrombocytopenia. Therefore, diagnosing t-TMA early and treating it appropriately is critical. The purpose of this study is to introduce the clinical characteristics and post-treatment course of patients who experienced t-TMA at our trauma center. Our aim is to aid physicians in the timely diagnosis and administration of appropriate treatment, which will ultimately improve patient prognoses.

## 2. Materials and Methods

### 2.1. Data Collection

We retrospectively reviewed 30 patients diagnosed with t-TMA among 2941 patients treated for acute trauma in the trauma intensive care unit of the Level I Trauma Center, Kyungpook National University Hospital, from January 2014 to December 2019. Data were collected on demographics, mechanisms of trauma, injury severity score (ISS), clinicopathological findings, treatment methods, complications, causes of death, volume of platelets transfused before therapeutic plasma exchange (TPE), laboratory findings, duration of renal replacement treatment (RRT), and duration of TPE.

### 2.2. Exclusion Criteria

This study excluded patients who were discharged or died within three days of admission, those who were under the age of 17, and those who experienced non-acute traumatic events, such as chronic subdural hematomas. Additionally, we excluded patients who were transferred to our trauma center from another institution after 24 h and patients diagnosed with chronic kidney disease.

### 2.3. Diagnostic Criteria of t-TMA

The diagnostic criteria for t-TMA in this study were as follows: (1) A history of recent acute traumatic events; (2) persistent thrombocytopenia (<50,000/µL) unresponsive to platelet transfusion post-complete hemostasis or absent recent bleeding; (3) hematologic findings indicative of microangiopathic hemolytic anemia (i.e., presence of schistocytes on peripheral blood smears, decreased haptoglobin, elevated lactate dehydrogenase (LDH), increased bilirubin levels in serum tests); and (4) exclusion of alternative conditions that may induce TMAs or generate schistocytes (e.g., disseminated intravascular coagulopathy [DIC], sepsis, drug-induced TMA, hemolytic uremic syndrome [HUS], or complement-mediated TMA).

### 2.4. Diagnostic Algorithm of t-TMA

After achieving complete hemostasis via conservative treatment as well as endovascular or surgical procedures, we would eliminate the possibility of DIC or sepsis if the platelet count was maintained below 50,000/µL for three days, with no response to continuous platelet transfusion. Initially, t-TMA was suspected in cases with normal laboratory profiles that did not indicate DIC or sepsis and when platelet transfusion was restricted unless deemed essential. Next, we would implement immediate evaluations of LDH and peripheral blood (PB) smear levels, with t-TMA strongly suspected if the LDH level was higher than normal (<250 U/L) and particularly if it was increasing compared with the previous test. If schistocytes were detected in the peripheral blood smear, various additional tests would be conducted to eliminate alternative etiologies. Conducting a Coombs test would rule out autoimmune or drug-induced hemolytic anemia; C3 and C4 levels would be measured to exclude complement-mediated TMA; conducting an anti-nuclear antibody test would exclude autoimmune disorders such as lupus nephritis and acute scleroderma; obtaining a stool culture would rule out HUS caused by a Shiga toxin-producing bacterial infection; evaluating a coagulation factor panel would exclude coagulation factor deficiencies; conducting a haptoglobin test would confirm hemolytic anemia; and assessing a disintegrin and metalloproteinase levels with thrombospondin motifs 13 (ADAMTS13) would confirm TTP. The diagnosis would then be verified by a hematologist, and methylprednisolone would be administered. Finally, after excluding all other potential causes of TMA, t-TMA would be confirmed, and TPE would commence.

### 2.5. Acute Renal Failure (ARF) Definition

In our study, ARF is described as acute renal failure requiring RRT, which corresponds to stage 3 in the urine output criteria of the RIFLE (Risk, Injury, Failure, Loss, and End-stage kidney disease) classification/staging system for acute renal failure (AKI) [<0.3 mL/kg per hour for 24 h or anuria for 12 h] [5].

### 2.6. Treatment Strategy of t-TMA

Upon diagnosis of t-TMA, platelet transfusion should be halted immediately unless it is unavoidable. The first-line treatment should be TPE with or without methylprednisolone, which should be administered as soon as possible. A patient would be treated solely with methylprednisolone if they refused TPE or were hemodynamically unstable. The endpoint of TPE was the moment at which the platelet count surpassed 150,000/µL, and the LDH level was normalized. RRT was initiated in patients with ARF in the form of continuous RRT, which was subsequently supplanted with conventional hemodialysis (HD) once the patient’s condition stabilized. Rituximab was used in cases where patients did not achieve remission with the initial treatment protocol of TPE and methylprednisolone or where patients recurred.

### 2.7. Statistical Analysis

Statistical analysis was conducted using SPSS for Windows, version 22.0 (SPSS, Inc., Chicago, IL, USA). Prior to analysis, data were assessed for normality using a Kolmogorov–Smirnov test. Continuous variables were reported as the mean ± standard deviation and categorical variables as absolute numbers and/or percentages. For comparisons, normally distributed continuous variables were analyzed using a Student’s *t*-test, whereas non-normally distributed variables were assessed with a Mann–Whitney U test. Categorical variables were examined using either a Pearson’s chi-squared test or Fisher’s exact test, as appropriate. The relationship between continuous variables was evaluated with the Pearson correlation coefficient, with r values indicating the strength and direction of the linear relationship (−1 to +1). A *p*-value of less than 0.05 was considered statistically significant. Normality was further verified with a Shapiro–Wilk test, and in instances where normality was not assumed, a Spearman’s rank correlation was utilized. All results are presented as the mean ± standard deviation, with analyses performed using two-tailed tests.

## 3. Results

### 3.1. Demography and Clinicopathologic Findings

**Figure 1:**
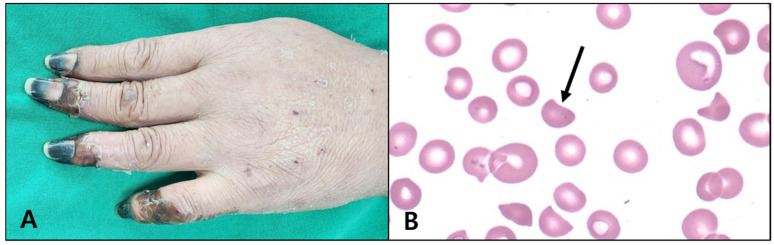
Clinical characteristics of patients with t-TMA. (**A**) Digital necrosis was observed in 13 patients (43.3%). (**B**) Schistocytes were identified in the peripheral blood smears of all patients, which is a characteristic finding of microangiopathic hemolytic anemia.

Between January 2014 and December 2019, 30 patients were diagnosed with t-TMA per the above-mentioned diagnostic criteria. Of these patients, 19 (63.3%) were men and 11 (36.7%) were women, and the mean age was 67.6 years. All 30 patients had experienced blunt-force trauma, and the average ISS was 23.0 ± 9.1 (range, 8–44). Regarding the clinical symptoms at the time of t-TMA diagnosis, digital necrosis was noted in 13 of the 30 patients (43.3%) (Figure 1A), alteration of mental status in 16 patients (53.3%), and non-infectious fever in 15 patients (50%). Regarding the laboratory findings at the time of diagnosis, total bilirubin levels were 6.3 ± 8.2 mg/dL (range, 1.05~41), LDH was 1541.9 ± 1067.5 U/L (range, 275~4117), BUN was 43.3 ± 30.9 mg/dL (range, 8.6~127.8), and creatinine was 2.6 ± 1.9 mg/dL (range, 0.5~8.0). Haptoglobin levels were measured in 26 patients and were very low (<10 mg/dL) in all of them. The ADAMTS13 test was performed on 25 patients with a mean of 61.4%. Of these, only one had ADAMTS13 activity ≤ 10% (diagnostic criteria of TTP [6]). Schistocytes were seen in all patients on PB smears, accounting for 3.9 ± 3.1% (range, 1~14%) of the examined fields (Figure 1B).

### 3.2. Onset Time of Clinical Findings

The first clinical finding was a platelet count of <50,000 without response to platelet transfusion, and the average onset was 1.1 ± 1.6 days (range, 0~7) after trauma. The next clinical finding was ARF with RRT starting at a mean of 1.9 ± 1.5 days (range, 0~7) after trauma.

### 3.3. AKI and RRT

**Figure 2:**
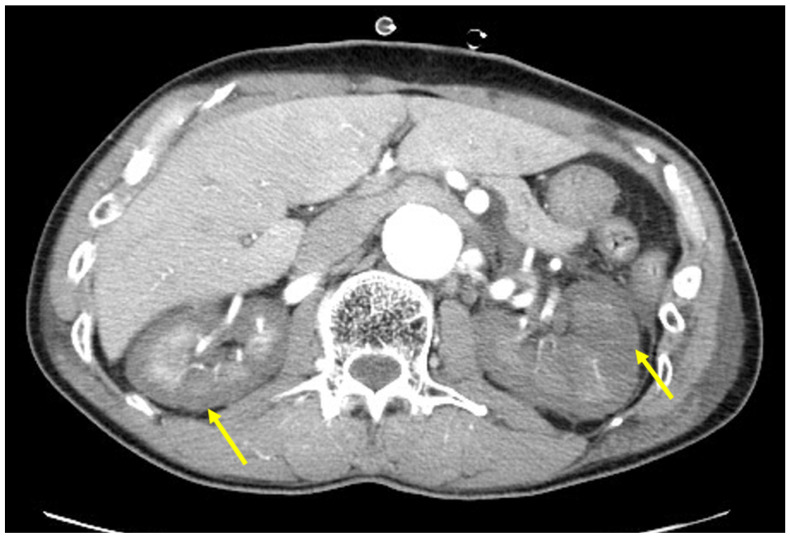
Contrast-enhanced abdominal CT of a t-TMA patient showing severely reduced renal perfusion (yellow arrow).

In total, 26 patients (89.7%) required RRT because of ARF (Figure 2). AKI completely resolved in 13 patients after a mean RRT period of 20.5 ± 16.1 days (range, 2~66). Factors affecting the RRT period were higher total bilirubin levels at the time of diagnosis (r = 0.643, *p* = 0.024) and lower ADAMTS13 activity (r = −0.659, *p* = 0.038), but RRT period was not affected by early TPE or the level of platelet transfusion before diagnosis. Of the 26 patients with AKI, eight died in hospital. Five of the remaining eighteen patients (5/18, 27.8%) developed chronic renal failure and had to undergo permanent hemodialysis. There were no factors affecting the transition to chronic renal failure. The clinical characteristics of t-TMA patients are summarized in Table 1.

### 3.4. Treatment and Response Rate

Eighteen patients were treated with TPE alone, seven with a TPE/methylprednisolone combination, and three with methylprednisolone alone. Remission was achieved in 29 patients (96.7%), with the one remaining patient achieving remission after using rituximab (CD20 monoclonal antibody). Two patients who did not respond to TPE subsequently relapsed while hospitalized but improved with rituximab.

### 3.5. TPE Duration

**Figure 3:**
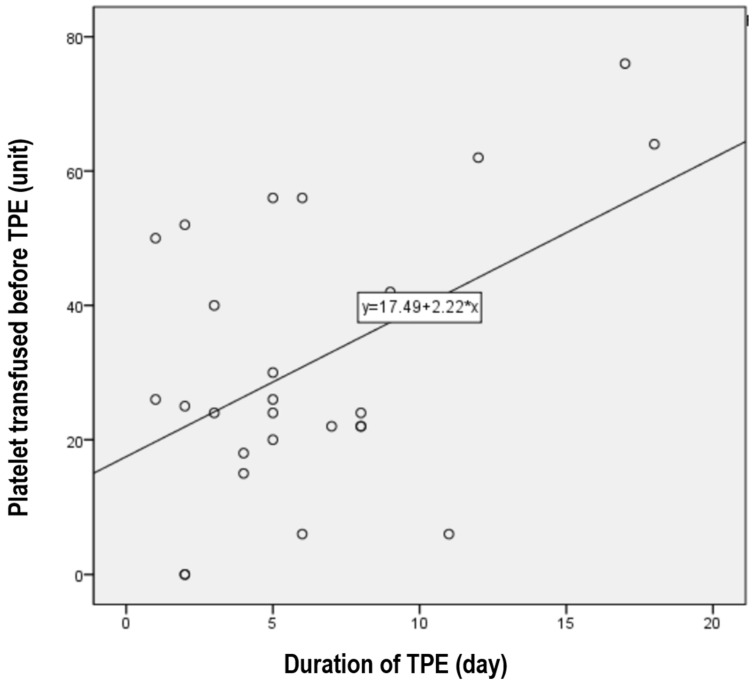
A positive relationship was observed between the amount of platelets transfused before TPE and the duration of TPE (r = 0.481, *p* = 0.013).

The mean TPE duration was 6.1 ± 4.4 days (range, 1~18), with a median of 5 days. The duration of TPE was longer in proportion to the number of platelets transfused before TPE (*r* = 0.481, *p* = 0.013) (Figure 3). However, there was no correlation with LDH, BUN, creatinine, proportion of schistocytes, ADAMTS13 activity, or early TPE.

### 3.6. Mortality

Eight patients died, resulting in a mortality rate of 26.7%. The most common cause of death was sepsis in five cases, followed by respiratory failure, acute MI, and cerebral infarct. Interestingly, four of the five patients who died from sepsis were treated with a TPE/methylprednisolone combination (Table 2). When patients were divided into two groups based on the presence or absence of sepsis and compared, methylprednisolone was found to affect the incidence of sepsis (Table 3).

## 4. Discussion

In 1952, Symmers [7] defined TMA as a pathologic condition that is distinguished by systemic microvascular thrombosis, anemia, thrombocytopenia, and target organ injury [1,2]. As TMA is a potentially fatal condition, it is considered a medical emergency. Platelet consumption and red blood cell disruption within the microvasculature can lead to consumptive thrombocytopenia and hemolytic anemia, which are almost always present in patients with TMA [8].

George and Nester categorized TMAs into nine disorders, referring to them as primary TMA syndromes that are characterized by evidence of a defined abnormality being the probable cause [2]. The selected terminology for these syndromes denotes their etiology, although prevalent nomenclatures such as TTP for ADMATS13-deficiency-induced TMA and HUS for Shiga toxin-induced TMA (ST-HUS) are nonetheless widely recognized. A causal abnormality, such as ADAMTS13 deficiency or a complement mutation, may remain asymptomatic until certain events trigger an acute TMA episode. These can include pregnancy, surgery, malignant hypertension, systemic infections, malignancies, autoimmune disorders, solid organ transplantation, or an inflammatory disorder [1,2]. However, recent studies have also reported TMA in surgical and trauma patients [3,9].

Chang describes TMA as a vascular microthrombotic disease, categorized into TTP and other TTP-like syndromes [10]. The most common group is TTP, which is caused by ADAMTS13 deficiency. However, a TTP-like syndrome has a different etiology, and although the mechanism for this has not been clearly identified, it is sometimes referred to as endotheliopathy [10]. Nevertheless, despite their differences, the clinical characteristics of a TTP-like syndrome and TTP are broadly similar, although there are currently no studies that have reported in detail the clinical aspects of t-TMA. Regardless of the initial cause, platelet transfusion not only exacerbates the progression of TMA but can also be fatal to the patient. Therefore, the clinical characteristics of t-TMA must be understood for prompt diagnosis and treatment.

In patients with severe trauma, if thrombocytopenia is accompanied by a decrease in hemoglobin, then consumptive thrombocytopenia should be suspected because of ongoing bleeding. Physicians will attempt to locate the bleeding focus. However, even if there is no recent bleeding, thrombocytopenia increases the risk of rebleeding or CNS bleeding, leading physicians to commence platelet transfusion. However, if the thrombocytopenia is caused by t-TMA, repeated platelet transfusions will only aggravate the microthrombosis, thereby worsening the patient’s condition. Therefore, it is imperative to consider the possibility that unexplained thrombocytopenia could be caused by t-TMA.

The first potential symptom of t-TMA is unexplained thrombocytopenia that persists despite repeated platelet transfusions. At this point, the patient should be free of recent bleeding. The next symptom is abrupt onset ARF, which appears almost simultaneously with thrombocytopenia. ARF is also characterized by the absence of preceding causes such as hypovolemic shock. Our data showed that 89.7% of all patients had a Stage 3 ARF of RIFLE classification that required RRT. On average, thrombocytopenia occurs 1.1 days after trauma, and ARF occurs after 1.9 days. Therefore, if ARF appears almost simultaneously alongside unexplained thrombocytopenia in a trauma patient, t-TMA should be suspected, and rapid diagnostic testing should be performed. Another characteristic finding of t-TMA is a progressive increase in LDH levels—a hemolysis marker that indicates ongoing injury to red blood cells within vessels that have been narrowed by microthrombosis [11]. LDH levels should decrease as t-TMA improves, meaning that the treatment’s endpoint is the normalization of LDH levels [12].

The biggest difference between t-TMA and TTP is the absence of ADAMTS13 deficiency. Only one of the patients in our study had an ADAMTS13 deficiency (activity, 0%). This patient was diagnosed with true TTP, which recurred during their hospitalization. At that time, the patient did not respond to TPE and was remitted with rituximab. Another difference from TTP is the frequency of AKI. In total, 89.7% of our study patients developed AKI sufficient to require RRT. This is a clear difference from TTP, where AKI occurs in only 10–27% of patients [13]. However, 53.3% and 50% of our t-TMA patients experienced altered mental states and fever, respectively, which was similar to the frequency in TTP patients (61% and 41%) [14]. Additionally, although the exact frequency of digital necrosis in TTP cases is unknown, it was reported in 43.3% of our t-TMA patients. The most characteristic finding of t-TMA, distinct from TTP, was the simultaneous occurrence of severe ARF alongside thrombocytopenia.

As mentioned above, the ADAMTS13 test is a very important test for differentiating between t-TMA and TTP and is also an essential test for identifying the characteristics of t-TMA. However, this study spanned six years, and during the early part of the study period, the ADAMTS13 test was not readily available in our hospital and had to be outsourced to an external testing agency. Given the urgency of trauma cases with suspected t-TMA and a lack of awareness among some center physicians at that time, ADAMTS13 testing was not performed for a few patients. However, we were able to conduct ADAMTS13 tests on 25 out of 30 patients (83.3%), and the results, except for one patient who was diagnosed with TTP, showed a relatively even distribution with a mean ± standard deviation activity level of 64.0 ± 23.2 (range, 28–106). Thus, the ADAMTS13 activity of t-TMA appears to be within the normal range.

The first step in treating both TMA and TTP is to perform TPE as soon as possible and before disease progression leads to irreversible organ dysfunction caused by tissue hypoxia [10]. However, if immediate TPE is not possible, high doses of methylprednisolone can be used as an alternative first-line treatment, as it can be effective as a combined therapy with TPE [15]. Unfortunately, both of these treatments require caution as they can lower the patient’s immunity and make them susceptible to infection [16]. This is well reflected by the fact that four out of the five patients who died of sepsis were treated with TPE and methylprednisolone combination therapy. Therefore, once treatment has commenced, it is recommended to up-stage prophylactic antibiotics, and more attention should be paid to infections.

Although many studies have analyzed the factors affecting the duration of TPE in TTP, there is a lack of research on TMA cases without TTP. In TTP patients, TPE duration can be influenced by disease severity, ADAMTS13 activity, infection, earlier initiation, and adjunctive therapy (rituximab) [17]. However, our data showed that only the number of platelets transfused before TPE affected its duration. ADAMTS13 activity and early TPE initiation had no effect, perhaps because the size of the patient group was too small to show any significant effect. However, considering the pathophysiology of TMA, platelet transfusion before treatment is contraindicated as it is expected to adversely affect disease progression [10]. Our results confirmed this.

Factors affecting the duration of RRT and conversion to CRF, such as early treatment and refraining from platelet transfusion, were expected to have a significant effect, but our data confirmed that this was not the case. However, the duration of RRT was found to be longer when ADAMTS13 activity was lower and bilirubin levels were higher, suggesting that the greater the disease severity, the longer the RRT.

Although there are statistical limitations due to the small sample size and reliance on univariate analysis, the analysis of factors influencing patient mortality revealed that the frequency of sepsis and the use of TPE/prednisolone combination therapy were significantly higher in the non-survivor group. In contrast, mortality was not substantially influenced by the ‘interval between platelet count <50 k and treatment’, a variable that reflects the expediency of treatment, and certain variables that indicate the severity of the disease. Additionally, the high frequency of prednisolone use in the sepsis group suggests that high-dose prednisolone may have some impact on infection risk. To assess the efficacy and hazards of prednisolone use for t-TMA, future large-scale prospective studies are required.

This study has some limitations. First, the relatively small sample size may limit the generalizability of the findings, although it provides valuable insights into the rare condition of t-TMA. Future studies should consider a multi-center approach or larger patient cohorts. Second, a retrospective study has the potential for confounding factors and selection bias during data collection. Prospective cohort studies in the future will help mitigate these limitations and provide more precise findings. Third, this study included patients with a diverse range of trauma mechanisms, which introduces heterogeneity that may affect the generalizability of some conclusions. The sample size was too small to allow for the analysis of subgroups. Fourth, the follow-up period in this study may not have been sufficient to evaluate the long-term outcomes of patients, particularly with regard to chronic kidney failure or long-term complications. Longer follow-up periods are necessary to fully understand the prognosis of patients with t-TMA.

## 5. Conclusions

This research has underlined the need for physicians to maintain a high index of suspicion for t-TMA in trauma patients presenting with unexplained thrombocytopenia, ARF, and hematologic findings consistent with microangiopathic hemolytic anemia. Timely interventions can significantly improve patient outcomes. Future studies with larger sample sizes are warranted to further elucidate the clinical characteristics, optimal treatment protocols, and long-term prognosis of t-TMA.

## Figures and Tables

**Table 1:** Clinical characteristics of t-TMA patients.

No.	Sex	Age	ISS	Hospital Stay	Trauma Mechanism	Diagnosis of Trauma	CRRT	To CRF	TPE	Methylprednisolone	Expire	Cause of Death
1	M	83	25	25	pedestrian TA	pelvic Fx	+		+	+	+	sepsis
2	M	76	27	8	passenger TA	mesenteric injury, SAH, MRF	+		+		+	MOF, cerebral infarction
3	M	86	4	29	pedestrian TA	pelvic Fx	+		+		+	acute MI
4	M	89	43	24	pedestrian TA	pelvic Fx	+			+		
5	M	77	27	20	motorcycle TA	spleen injury, skull Fx	+		+	+	+	pneumonia, sepsis
6	F	84	16	52	pedestrian TA	pelvic, extremity Fx	+		+			
7	F	87	9	85	pedestrian TA	extremity Fx, popliteal artery injury	+		+	+		
8	F	74	26	51	pedestrian TA	extremity Fx, diaphragmatic rupture	+		+		+	neutropenia, sepsis
9	F	74	22	66	passenger TA	spine Fx, MRF	+		+	+	+	pneumonia, sepsis
10	M	63	38	73	motorcycle TA	mesenteric injury, aorta injury, extremity Fx	+	+	+			
11	M	23	22	71	motorcycle TA	liver injury	+		+			
12	M	48	41	208	pedestrian TA	liver injury, pelvic Fx	+		+			
13	F	94	14	4	fall	retroperitoneal hematoma, spine Fx	+		+	+	+	refusal of mechanical ventilator
14	M	18	24	59	motorcycle TA	stomach rupture, spleen injury, small bowel injury	+		+			
15	M	61	13	145	passenger TA	extremity Fx	+		+			
16	M	78	8	71	pedestrian TA	liver/spleen injury, pelvic Fx			+			
17	F	80	22	44	fall	pelvic Fx, bladder injury	+		+			
18	F	77	13	44	pedestrian TA	pelvic Fx	+		+			
19	F	87	22	70	pedestrian TA	pelvic Fx, MRF, facial Fx	+	+	+	+		
20	M	65	29	88	pedestrian TA	MRF, diaphragmatic rupture	+		+			
21	M	57	25	37	pedestrian TA	kidney/spleen injury	+	+	+			
22	M	89	21	36	fall	pelvic Fx				+		
23	M	58	22	97	passenger TA	extremity Fx	+	+		+		
24	M	70	16	72	slip	ICH	+		+	+		
25	F	21	34	209	fall	spleen/kidney/mesenteric injury, pelvic Fx, MRF	+		+			
26	M	58	33	65	passenger TA	mesenteric injury, heart valve rupture	+	+	+			
27	M	54	24	43	passenger TA	spleen injury, MRF	+		+			
28	M	74	25	58	pedestrian TA	spleen/liver injury, diaphragmatic rupture			+	+		
29	F	53	29	23	fall	pelvic Fx, aorta injury, liver injury	+		+	+	+	sepsis
30	F	71	17	65	pedestrian TA	pelvic Fx			+			

ISS: injury severity score; CRRT: continuous renal replacement therapy; CRF: chronic renal failure; TPE: therapeutic plasma exchange; M: male; F: female; TA: traffic accident; Fx: fracture; SAH: sub-arachnoid hemorrhage; MRF: multiple rib fracture; MOF: multiple organ failure; MI: myocardial infarction; ICH: intra-cerebral hemorrhage.

**Table 2:** Comparison between survivor and non-survivor groups.

Variable	Survivor (n = 22)	Non-Survivor (n = 8)	*p*-Value
Sex (male) ^a^	15 (68.2)	4 (50.0)	0.417
Age ^b^ (year)	64.1 ± 21.5	77.1 ± 11.9	0.119
ISS ^b^	23.5 ± 9.7	21.8 ± 8.5	0.656
Fever ^a^	13 (59.1)	2 (25.0)	0.215
Digital necrosis ^a^	9 (40.9)	4 (50.0)	0.485
Total bilirubin ^b^ (mg/dL)	5.6 ± 8.6	8.2 ± 7.1	0.445
LDH ^b^ (U/L)	1622.1 ± 1105.8	1321.3 ± 988.4	0.504
ADAMTS13 activity ^b^ (%)	62.9 ± 27.9	58.1 ± 24.7	0.677
Schistocytes ^b^ (%)	3.7 ± 3.4	4.4 ± 2.1	0.621
TPE duration ^b^ (day)	6.1 ± 4.6	5.5 ± 4.5	0.754
Interval between Plt < 50 k and Tx ^b^ (day)	3.7 ± 2.1	3.4 ± 2.6	0.703
Transfused Plt before Tx ^b^(unit)	29.4 ± 19.1	31.1 ± 21.9	0.835
Sepsis ^a^	0 (0)	5 (62.5)	0.000
TPE + methylprednisolone ^a^	4 (18.2)	5 (62.5)	0.032
TPE ^a^	19 (86.3)	8 (100)	0.271
Methylprednisolone ^a^	7 (31.8)	5 (62.5)	0.210
Empirical antibiotics ^a^	12 (54.5)	3 (37.5)	0.682

^a^ Values are presented as n (%). ^b^ Values are presented as the mean ± standard deviation. ISS: injury severity score; LDH: lactate dehydrogenase; ADAMTS13: a disintegrin and metalloproteinase with thrombospondin motifs 13; TPE: therapeutic plasma exchange; Tx: treatment.

**Table 3:** Comparison between sepsis and non-sepsis groups.

Variable	Sepsis (n = 5)	Non-Sepsis (n = 25)	*p*-Value
Sex (male) ^a^	2 (40)	17 (68)	0.327
Age ^b^ (year)	72.2 ± 11.3	66.7 ± 21.5	0.584
ISS ^b^	25.8 ± 2.6	22.5 ± 10.1	0.475
Fever ^a^	1 (20)	14 (56)	0.142
Digital necrosis ^a^	3 (60)	10 (40)	0.628
Total bilirubin ^b^ (mg/dL)	9.5 ± 8.7	5.7 ± 8.1	0.352
LDH ^b^ (U/L)	1473.6 ± 1271.3	1555.6 ± 1051.9	0.879
ADAMTS13 activity ^b^ (%)	54.0 ± 18.9	63.3 ± 28.1	0.497
Schistocytes ^b^ (%)	3.8 ± 1.1	3.9 ± 3.4	0.939
TPE duration ^b^ (day)	7.2 ± 4.7	5.6 ± 4.5	0.490
Interval between Plt < 50 k and Tx ^b^ (day)	3.2 ± 2.5	3.7 ± 2.2	0.636
Transfused Plt before Tx ^b^ (unit)	26.6 ± 21.3	30.5 ± 19.5	0.689
TPE + methylprednisolone^a^	4 (80)	5 (20)	0.019
TPE ^a^	5 (100)	22 (88)	1.000
Methylprednisolone ^a^	4 (80)	8 (32)	0.046
Empirical antibiotics ^a^	2 (40)	13 (52)	1.000

^a^ Values are presented as n (%). ^b^ Values are presented as the mean ± standard deviation. ISS: injury severity score; LDH: lactate dehydrogenase; ADAMTS13: a disintegrin and metalloproteinase with thrombospondin motifs 13; TPE: therapeutic plasma exchange; Tx: treatment.

## Data Availability

The data presented in this study are available upon request from the corresponding author. The data are not publicly available due to privacy or ethical restrictions.
